# Supplementary material for: The Filippi’s Glands of Giant Silk Moths: To Be or Not to Be?
Source: Insects. 2021 Nov 19;12(11):1040. doi: 10.3390/insects12111040 (PMC8619679; doi:10.3390/insects12111040)
Supplement: Supplementary file 1 [file insects-12-01040-s001.zip › insects-1450041-supplementary.pdf]

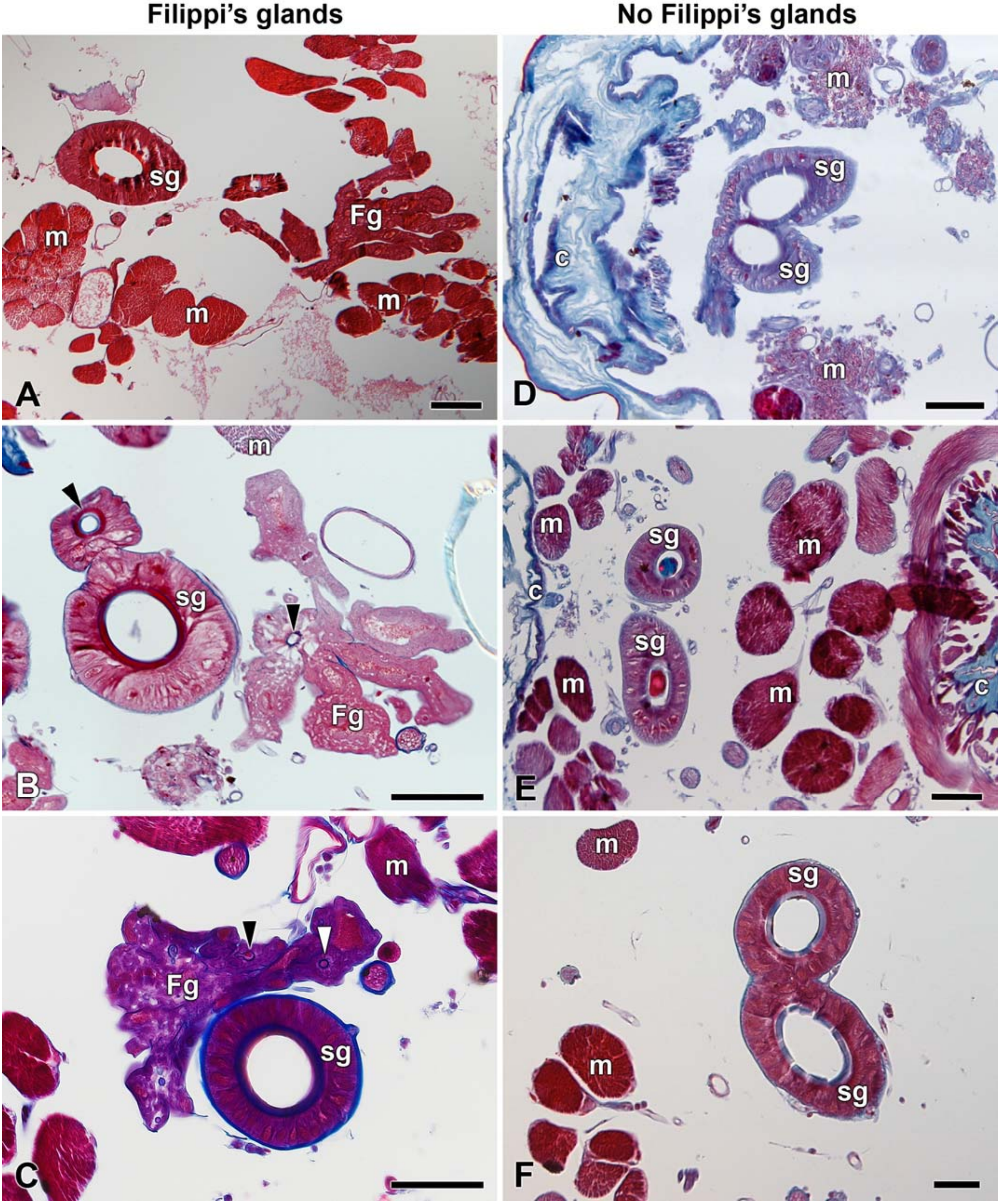

Figure S1. Cross-sections through the spinning apparatus of last silk moth larval instars labeled by Masson trichrome stain. (**A**) The polyphemus moth, *Antheraea polyphemus*. (**B**) The Indian moon moth, *Actias selene*. (**C**) The small emperor moth, *Saturnia pavonia*. (**D**) The cecropia silk moth, *Hyalophora cecropia*. (**E**) The Eri silk moth, *Samia cynthia*. (**F**) The Atlas moth, *Attacus atlas*. Note that the FGs in *H. cecropia*, *S. cynthia*, and *A. atlas* are missing. The arrowheads indicate the ducts of FGs. Abbreviations:

cuticle (c), Filippi's gland (Fg), muscles of the silk press (m), silk gland (sg), and silk press of the spinning apparatus (spr). Scale bars: 100 μm.

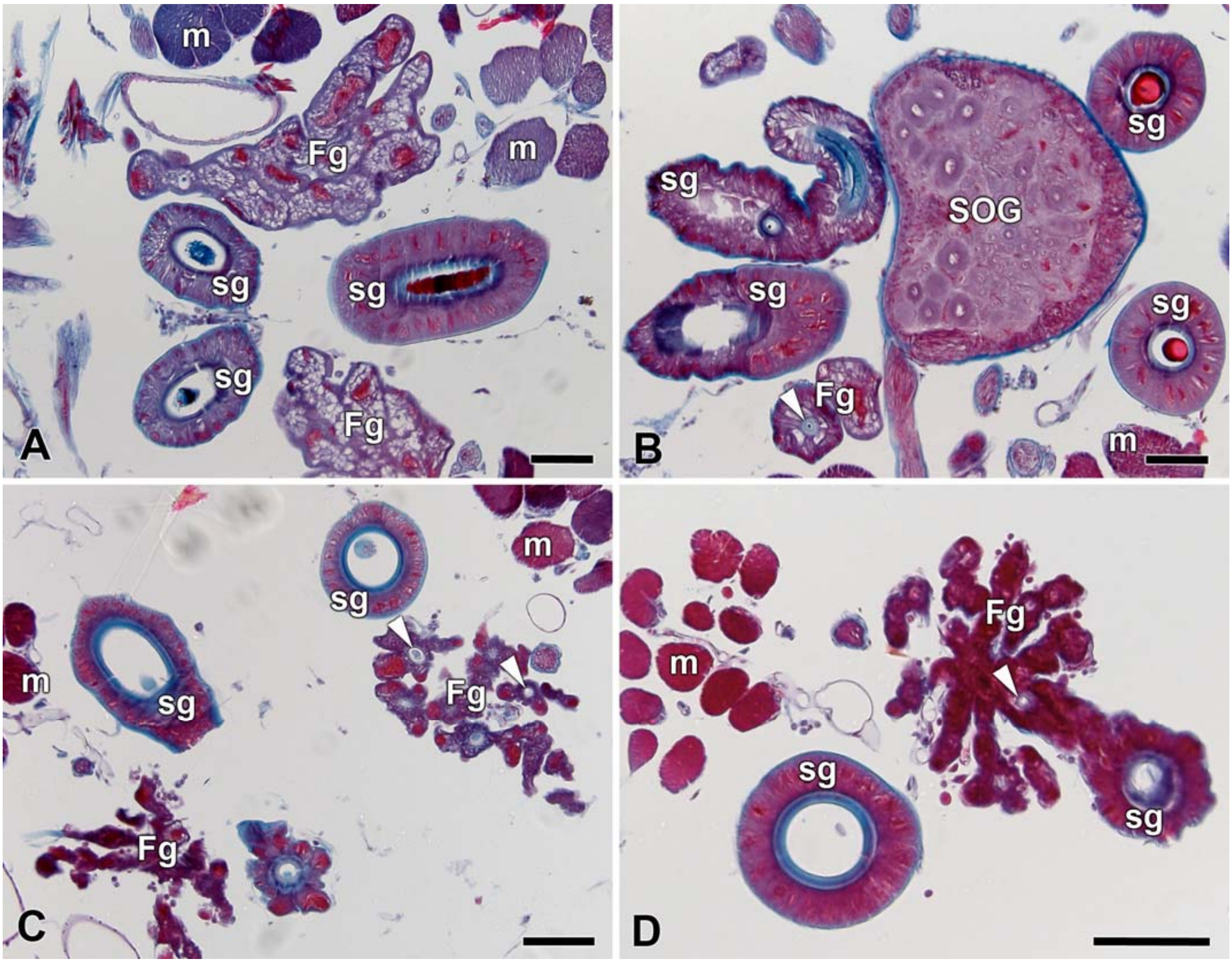

Figure S2. Cross-sections through the spinning apparatus of last silk moth larval instars labeled by Masson's trichrome stain. (**A**,**B**) The Chinese oak silk moth, *Antheraea pernyi.* (**C**,**D**) The Japanese oak silk moth, *Antheraea yamamai.* The arrowheads indicate the ducts of FGs. Abbreviations: Filippi's gland (Fg), muscles of the silk press (m), silk gland (sg), and suboesophageal ganglion (SOG). Scale bars: 100 μm.

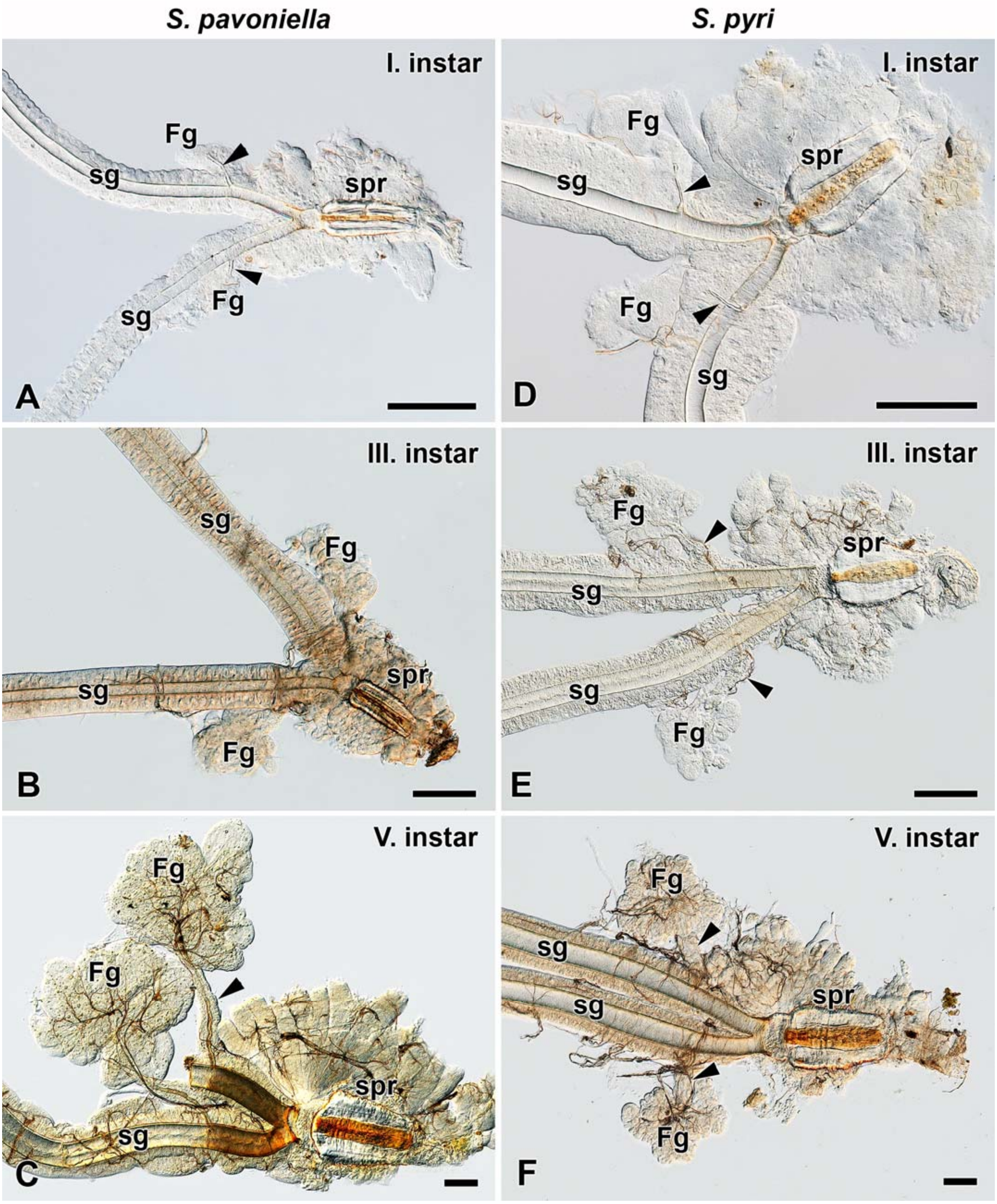

Figure S3. Development of spinning apparatuses of Saturniidae, 1st, 3rd, and 5th larval instars. (**A**–**C**) The Ligurian emperor moth, *Saturnia pavoniella*. (**D**–**F**) The giant peacock moth, *Saturnia pyri*. The arrowheads indicate the ducts of the FGs entering the lumen of the SGs. Abbreviations: Filippi's gland (Fg), silk gland (sg), and silk press of the spinning apparatus (spr). Scale bars: 100 μm.

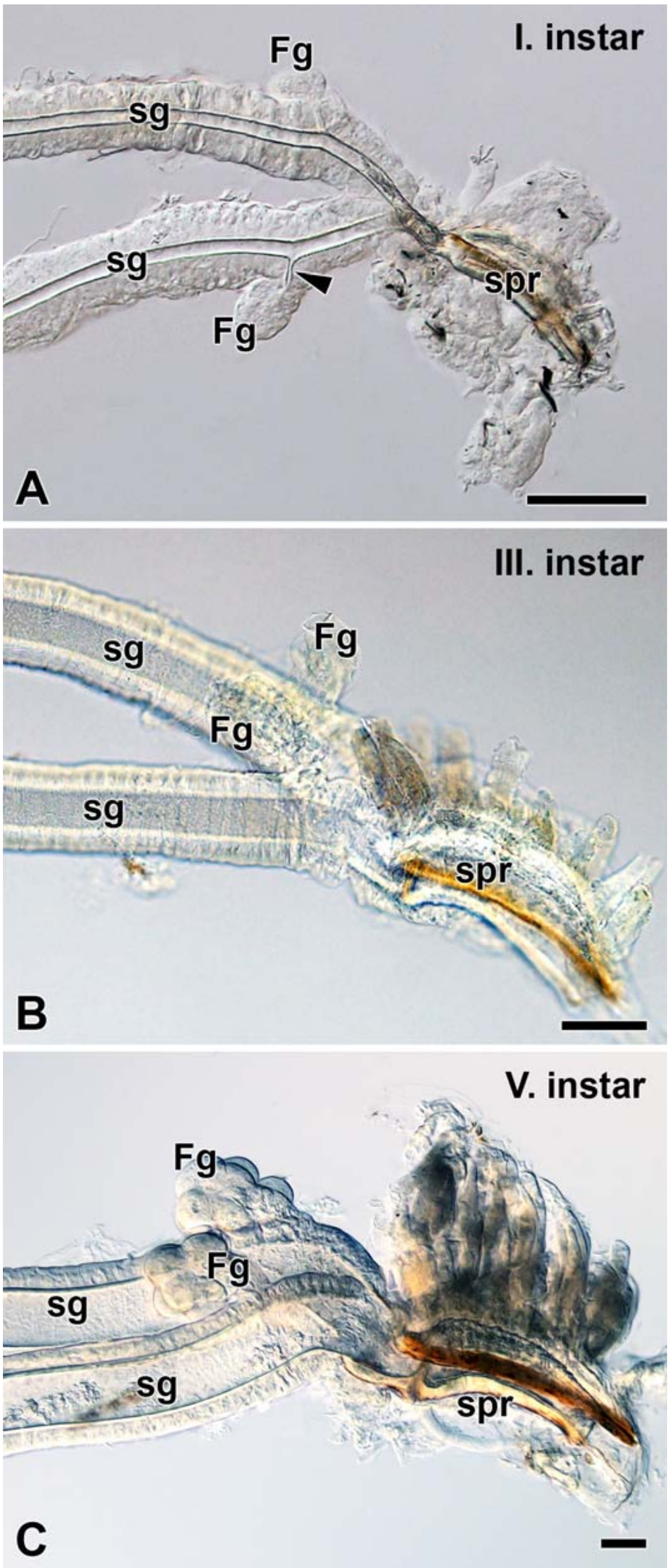

Figure S4. Development of spinning apparatus of *Antherina suraka* (Saturniidae). (**A**) First larval instar. (**B**) Third larval instar. (**C**) Fifth larval instar. The arrowhead indicates the duct of the FG entering the lumen of the SG. Abbreviations: Filippi's gland (Fg), silk gland (sg), and silk press of the spinning apparatus (spr). Scale bars: 100 μm.

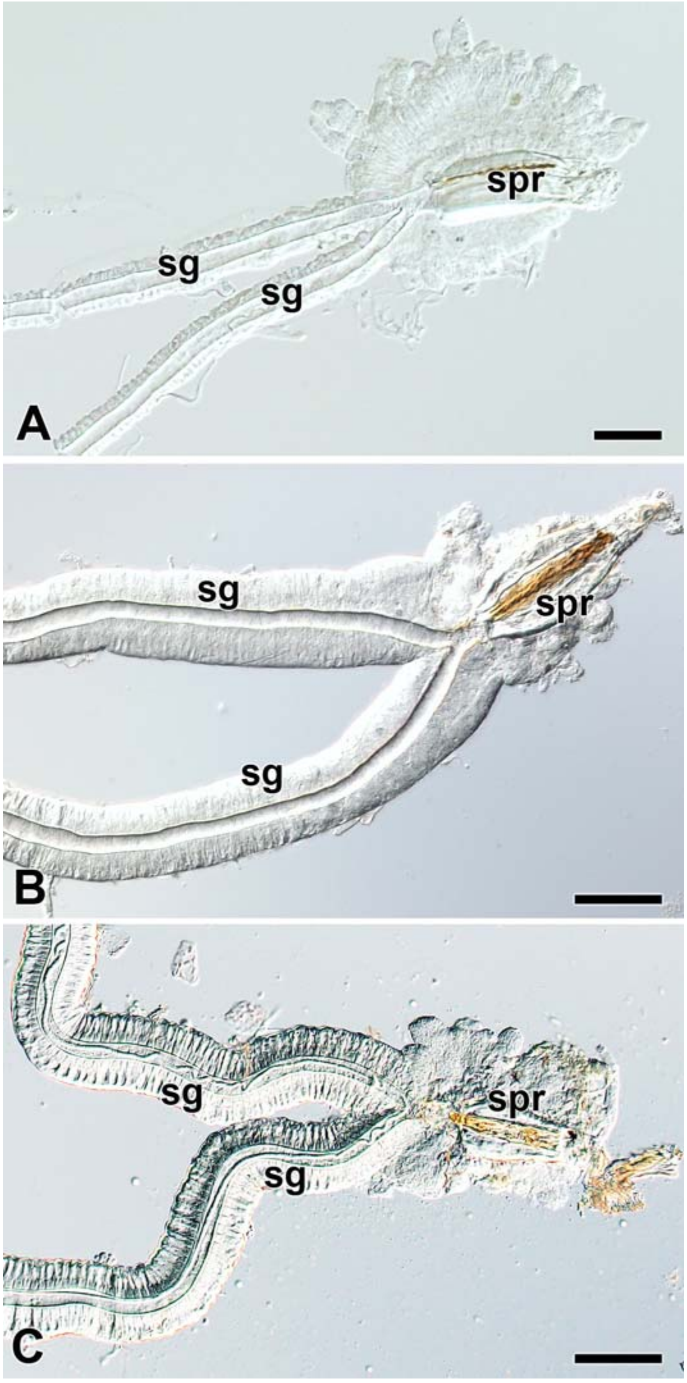

Figure S5. Spinning apparatuses of the first larval instar of Saturniidae. (**A**) The promethea silk moth, *Callosamia promethea.* (**B**) The African emperor moth, *Nudaurelia krucki.* (**C**) The tau emperor moth, *Aglia tau*. Note that in all three species the FGs are missing. Abbreviations: silk gland (sg) and silk press of the spinning apparatus (spr). Scale bars: 100 μm.

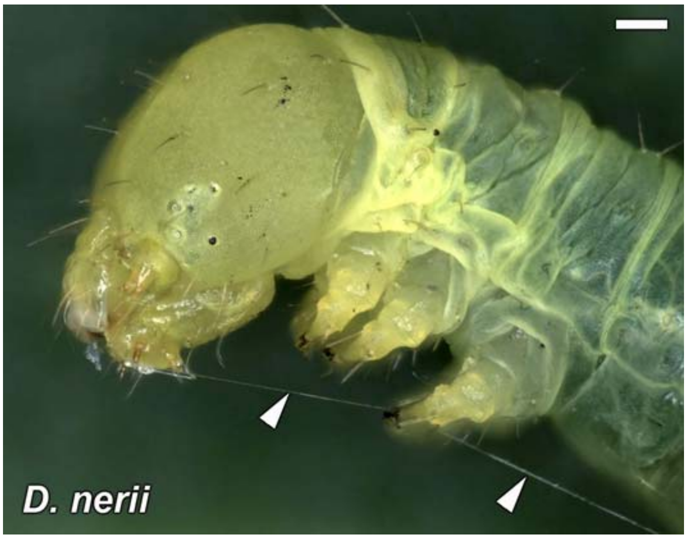

Figure S6. Rescue silk fiber of the *Daphnis nerii* 1st larval instar. Arrowheads depict the extruding rescue Scheme 250 μm.
